# Healthcare personnel absenteeism, presenteeism, and staffing challenges during epidemics

**DOI:** 10.1017/ice.2020.453

**Published:** 2020-10-26

**Authors:** Douglas W. Challener, Laura E. Breeher, JoEllen Frain, Melanie D. Swift, Pritish K. Tosh, John O’Horo

**Affiliations:** 1 Division of Infectious Diseases, Mayo Clinic, Rochester, Minnesota; 2 Preventive, Occupational and Aerospace Medicine, Mayo Clinic, Rochester, Minnesota; 3 Human Resources, Mayo Clinic, Rochester, Minnesota; 4 Division of Pulmonary and Critical Care Medicine, Mayo Clinic, Rochester, Minnesota

## Abstract

**Objective:**

Presenteeism is an expensive and challenging problem in the healthcare industry. In anticipation of the staffing challenges expected with the COVID-19 pandemic, we examined a decade of payroll data for a healthcare workforce. We aimed to determine the effect of seasonal influenza-like illness (ILI) on absences to support COVID-19 staffing plans.

**Design:**

Retrospective cohort study.

**Setting:**

Large academic medical center in the United States.

**Participants:**

Employees of the academic medical center who were on payroll between the years of 2009 and 2019.

**Methods:**

Biweekly institutional payroll data was evaluated for unscheduled absences as a marker for acute illness-related work absences. Linear regression models, stratified by payroll status (salaried vs hourly employees) were developed for unscheduled absences as a function of local ILI.

**Results:**

Both hours worked and unscheduled absences were significantly related to the community prevalence of influenza-like illness in our cohort. These effects were stronger in hourly employees.

**Conclusions:**

Organizations should target their messaging at encouraging salaried staff to stay home when ill.

Presenteeism, the act of attending work while ill, is a source of reduced productivity for employers, especially in healthcare.^1^ Investigations of presenteeism as it relates to chronic health conditions have found that its costs greatly exceed those of absenteeism.^2^ In one survey, nearly 60% of resident physicians answered that they had worked while ill at least once in the past year; residents later in training were more likely to come to work ill.^3^ In healthcare, presenteeism has also been associated with adverse patient safety and quality of care outcomes.^4^ The term “infectious presenteeism” has been coined to describe presenteeism in the face of an infectious disease. There are many examples of healthcare providers whose presence at work while ill resulted in the infection of patients.^5-7^ In self-reported survey data, healthcare providers have reported working while ill with influenza-like illness (ILI).^8^ A number of underlying causes for this behavior have been suggested, including a strong sense of duty to care for patients, disciplinary actions for missed work, and the lack of universal paid sick leave in the United States.^9,10^ Economic instability during pandemics as well as demand for healthcare services to care for ill patients may further exacerbate this problem.

The emergence of coronavirus disease 2019 (COVID-19) in late 2019 has posed a challenge for healthcare facilities to maximize the productivity of the healthcare workforce through anticipated patient surges while minimizing staff presenteeism during the pandemic. To prevent transmission to coworkers and patients, it is vital to support staff absences when workers are either ill or have been exposed to severe acute respiratory coronavirus virus 2 (SARS-CoV-2). To anticipate our challenges and to target messaging to healthcare workers, we examined historical payroll data for absenteeism from 2009 through 2019 for a healthcare workforce in Rochester, Minnesota. We obtained the Minnesota incidence of influenza-like illness (ILI) during these years from the Minnesota Department of Health and evaluated these data for patterns in healthcare absenteeism to guide approaches to minimize presenteeism during the current COVID-19 pandemic.

## Methods

Biweekly institutional payroll data for the years from 2009 through 2019 were obtained. This data set contained hours worked, unscheduled absences, scheduled paid time off, and full-time equivalent staff for the workforce at a large academic medical center in Rochester, Minnesota, broken down by payroll status (salaried vs hourly). Data for ILI activity (defined as the estimated percent of total outpatient visits attributed to ILI in a given week) in Minnesota were obtained from the publicly available Centers for Disease Control (CDC) and Minnesota Department of Health ILI data sets.^11^

Linear regression models, stratified by pay status (salaried or hourly), were developed for unscheduled absences as a function of ILI in Minnesota. To estimate the magnitude of unscheduled absences, hours of unscheduled paid time off were added to other unscheduled absence hours and divided by the total number of paid hours for each pay period. The same payroll time codes were used for both salaried and hourly workers. These codes are reported the same way regardless of employee salaried status except that salaried staff must report absences in multiples of full or half shifts. Unscheduled absences were then used as a marker for acute illness-related work absences. To assess the relationship between the number of working hours and ILI, biweekly ratios of working hours to total paid hours were calculated. Univariable linear regression models, stratified by payroll status, were developed to predict this measure as a function of ILI in Minnesota.

## Results

**Fig. 1.**
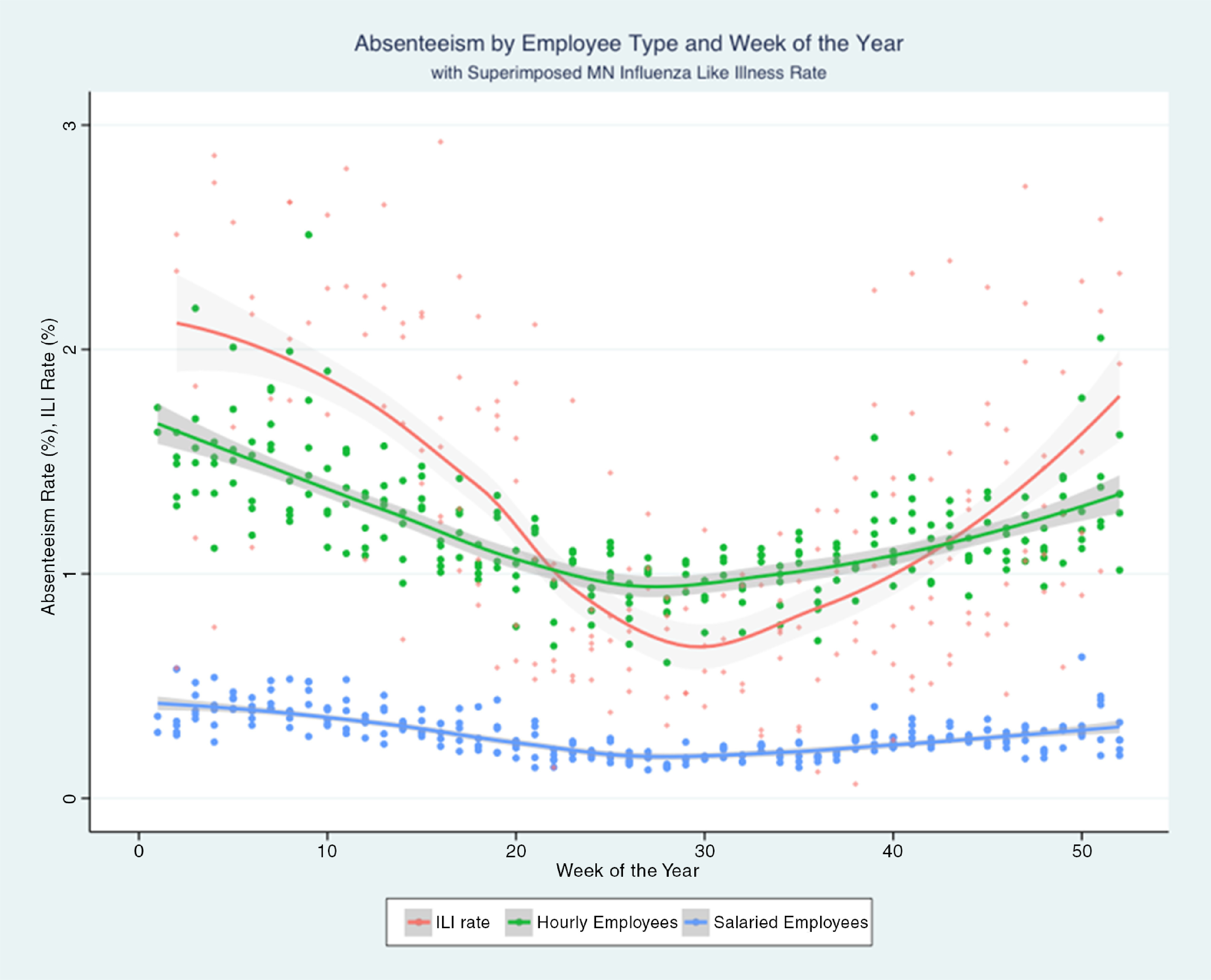
Rate of unscheduled absences compared to total hours worked and influenza-like activity in Minnesota over the calendar year. Each point represents one 2-week pay period.

An analysis of biweekly payroll data between December 23, 2009, and December 25, 2019, (254 pay periods) showed that, in both salaried and hourly employees, as ILI increases, the proportion of all absence hours that are unscheduled increases (Fig. 1). ILI is a statistically significant predictor of unscheduled absences in both salaried and hourly workers (*P* < .01). However, the effect is different in the 2 groups: salaried employees have fewer documented unscheduled absences than hourly workers (*P* < .01). For every 1% increase in ILI in the population of the state, hourly workers have a 0.14% increase in the percentage of unscheduled absence hours. This effect is lower in salaried workers, with an increase of 0.04% of unscheduled absences for every 1% increase in ILI in the state.

In addition to increased unscheduled absence rates with increased ILI, the proportion of paid hours in which the employees are working increases. For every increase in ILI by 1%, the proportion of paid hours that are worked increases by 0.2% in hourly workers (*P* = .04). We did not detect a statistically significant linear relationship between ILI and proportion of paid hours that are worked by salaried employees.

## Discussion

The last 10 years of data for this cohort of healthcare personnel suggest that absenteeism and increased work hours correlate directly with the level of ILI in the community. These effects are greater for hourly healthcare personnel than for salaried healthcare personnel. Historically, the percentage of total paid hours the healthcare worker works has rarely fallen below 80%, even in the weeks of highest ILI activity during the year. Staffing was disproportionately low at times of low ILI prevalence. This finding is likely due to a high proportion of employees taking vacation during low ILI months in the Midwest (ie, summer) as well as increased staffing during times of high ILI activity.

Unscheduled absences as a percentage of total paid hours is likely a better measure of staff absence due to illness than scheduled absences. Both hourly and salaried workers have more unscheduled absences as ILI increases. Salaried employees take fewer unscheduled absences than hourly workers by a large proportion. This finding may seem counterintuitive because salaried workers have less financial incentive to come to work while ill. However, salaried employees may have more flexibility in adjusting job tasks to allow them to remain productive in non–patient-care activities or working from home despite mild illness. In addition, salaried employees in healthcare are often physicians and administrators, and they may feel more reluctant to take unscheduled absence and more likely to collaborate with colleagues to ensure that the work is safely accomplished. Previous research has shown that these 2 groups in healthcare are less likely to have sickness absences.^11^ Salaried employees may also be less likely to report unscheduled absences to payroll due to routinely working outside defined office hours.

A potential limitation of this study is the use of Minnesota statewide ILI data to apply to a healthcare workforce, which is located in southern Minnesota. To assess this possibility, we examined local lab positivity rates for influenza and found that they mirrored the state data, suggesting that Minnesota Department of Health data for the state is representative of local ILI activity. Also, correlation between ILI activity and staffing does not prove causation., and there are a number of potential reasons for unscheduled absences in addition to ILI, such as holidays, weather conditions, and academic schedules, that may contribute to seasonal fluctuations in healthcare staffing.

Our findings suggest that methods are in place to accommodate flexible staffing to increase capacity during influenza season. During a pandemic such as COVID-19, institutional efforts to prevent infectious presenteeism are necessary. This messaging should be targeted to all employees, but it may have the most benefit among staff members who are salaried, given the lower routine rate of unscheduled absences in this group. Emphasizing the importance of this message and reducing the number of staff who attend work while ill should minimize the effects of contagious infectious diseases on healthcare organizations.

## Applications to COVID-19

Our analysis has shown that hourly workers are more likely to take unscheduled time away for acute illness compared to salaried employees. As ILI increases, the total number of hours worked for hourly workers increases reflective of the institution’s proactive staffing to accommodate both patient needs and time away for ill staff. The ability to adjust workforce hours has been implemented on a larger scale with COVID-19 due to illnesses among healthcare personnel and the potential for postexposure quarantines. In addition, healthcare personnel have been encouraged to stay home if they are ill to preserve the overall health of the workforce.

At our institution, we used the knowledge gained from this analysis as well as input from multiple stakeholders to design a proactive approach to support staffing during the COVID-19 pandemic. Examples of actions taken include but are not limited to the following:

- Developing nonpunitive institutional policies to support continued pay for staff who are ill to take time off or work from home without impact to pay and relaxing requirements that employees provide a doctor’s note to exercise sick leave
- Relaxing traditional attendance policies to provide greater flexibility for staff to stay home when ill
- Supporting appropriate quarantine of staff exposed to COVID-19, regardless of whether the exposure was from a patient or a household or community contact, to avoid transmission within the healthcare setting
- Planning for surges to ensure appropriate staffing, accounting for staff becoming ill and unscheduled absences being higher than typical
- Identifying creative ways to allow telework for staff who do not typically work from home
- Training staff for different tasks to allow redeployment within the organization
- Increasing virtual connections including patient visits and meetings.

Moreover, discouraging presenteeism and encouraging appropriate absenteeism will require a cultural shift. Healthcare workers feel a duty to patients and their coworkers to work whenever possible, so physicians, managers, and other healthcare leaders will need to model this behavior to support sustainable health and to minimize disease impact to the healthcare workforce. During a pandemic such as COVID-19, institutional efforts to prevent infectious presenteeism are necessary. This messaging should be targeted to all employees but may have the most impact in salaried staff members given the lower rate of unscheduled absences in this group. Emphasizing the importance of this message and reducing the number of staff who attend work while ill should minimize the effects of contagious infectious diseases on healthcare organizations. Unscheduled absences for ill staff have an immediate negative impact on staffing, and this practice has a long-term positive impact because fewer staff contract the infectious disease. Within our own staff, we have observed a profound willingness to be flexible in working hours and tasks to meet patients’ needs. This determination to care for patients and work additional hours combined with institutional planning will help to minimize presenteeism and its impacts throughout the country.

## References

[1] Rantanen I , Tuominen R . Relative magnitude of presenteeism and absenteeism and work-related factors affecting them among health care professionals. Int Arch Occ Env Hea 2011;84:225–230. 10.1007/s00420-010-0604-5 21140162

[2] Collins JJ , Baase CM , Sharda CE , et al. The assessment of chronic health conditions on work performance, absence, and total economic impact for employers. J Occup Environ Med 2005;47:547–557. 15951714 10.1097/01.jom.0000166864.58664.29

[3] Jena AB , Baldwin DC , Daugherty SR , Meltzer DO , Arora VM . Presenteeism among resident physicians. JAMA 2010;304:1166–1168. 20841527 10.1001/jama.2010.1315

[4] Letvak SA , Ruhm CJ , Gupta SN . Nurses’ presenteeism and its effects on self-reported quality of care and costs. Am J Nurs 2012;112:30–38. 10.1097/01.NAJ.0000411176.15696.f9 22261652

[5] Widera E , Chang A , Chen HL . Presenteeism: a public health hazard. J Gen Intern Med 2010;25:1244–1247. 20549378 10.1007/s11606-010-1422-x PMC2947637

[6] Ward A , Caro J , Bassinet L , Housset B , O’Brien JA , Guiso N . Health and economic consequences of an outbreak of pertussis among healthcare workers in a hospital in France. Infect Control Hosp Epidemiol 2005;26:288–292. 15796282 10.1086/502541

[7] Albrich WC , Harbarth S . Healthcare workers: source, vector, or victim of MRSA? Lancet Infect Dis 2008;8:289–301. 18471774 10.1016/S1473-3099(08)70097-5

[8] Chiu S , Black CL , Yue X , et al. Working with influenza-like illness: presenteeism among US health care personnel during the 2014–2015 influenza season. Am J Infect Control 2017;45:1254–1258. 28526310 10.1016/j.ajic.2017.04.008 PMC5670002

[9] Pichler S , Ziebarth NR . The pros and cons of sick pay schemes: testing for contagious presenteeism and noncontagious absenteeism behavior. J Public Econ 2017;156:14–33.

[10] Landry M , Miller C . Presenteeism: are we hurting the patients we are trying to help? J Gen Intern Med 2010;25:1142–1143. 20740323 10.1007/s11606-010-1487-6 PMC2947647

[11] Weekly influenza and respiratory activity: statistics. Minnesota Department of Health website. https://www.health.state.mn.us/diseases/flu/stats/index.html. Accessed February 15, 2020.

[12] Gorman E , Yu S , Alamgir H . When healthcare workers get sick: exploring sickness absenteeism in British Columbia, Canada. Work 2010;35:117–123. 20164606 10.3233/WOR-2010-0963

